# Patient factors associated with conveyance decision-making by Emergency Medical Services professionals in patients with a syncope: a cross-sectional factorial survey design

**DOI:** 10.1186/s12873-023-00890-y

**Published:** 2023-10-05

**Authors:** B. Bastiaan A. Ort, Lucia G. uit het Broek, Harm de Bruin, Reinier P. Akkermans, Ben Goosselink, Hester Vermeulen, Remco H.A. Ebben, Lilian C.M. Vloet, Sivera A.A. Berben

**Affiliations:** 1 School of Health Studies, Research Department of Emergency and Critical Care, HAN University of Applied Sciences, Nijmegen, The Netherlands; 2 Emergency Medical Service, RAV Utrecht, Utrecht, The Netherlands; 3 Radboud Institute for Health Sciences, Scientific Institute for Quality of Healthcare, Radboud University Medical Centre , Nijmegen, The Netherlands; 4 Radboud Institute for Health Sciences, Department of Primary and Community Care, Radboud University Medical Centre, Nijmegen, The Netherlands; 5 Emergency Medical Service, Ambulance IJsselland, Zwolle, The Netherlands; 6 Emergency Medical Service, Public Health and Safety region Gelderland-Midden, Arnhem, The Netherlands

**Keywords:** Ambulance care, Emergency medical services, Transient loss of consciousness, Vignette study

## Abstract

**Background:**

The clinical decision-making of non-conveyance is perceived as complex and difficult by emergency medical services (EMS) professionals. Patients with a transient loss of consciousness (TLOC) based on syncope constitute a significant part of the non-conveyance population. Risk stratification is the basis of the clinical decision-making process by EMS professionals. This risk stratification is based on various patient factors. This study aimed to explore patient factors significantly associated with conveyance decision-making by EMS professionals in patients with a TLOC based on syncope.

**Methods:**

A cross-sectional vignette study with a factorial survey design was conducted. Patient factors were derived from the “National Protocol Ambulance Care”, and all possible combinations of these factors and underlying categories were combined, resulting in 256 unique vignettes (2*4*4*4*2 = 256). Patient factors presented either low-risk or high-risk factors for adverse events. Data were collected through an online questionnaire, in which participants received a random sample of 15 vignettes. For each vignette, the respondent indicated whether the patient would need to be conveyed to the emergency department or not. A multilevel logistic regression analysis with stepwise backward elimination was performed to analyse factors significantly associated with conveyance decision-making. In the logistic model, we modelled the probability of non-conveyance.

**Results:**

110 respondents were included, with 1646 vignettes being assessed. Mean age 45.5 (SD 9.3), male gender 63.6%, and years of experience 13.2 (SD 8.9). Multilevel analysis showed two patient factors contributing significantly to conveyance decision-making: ‘red flags’ and ‘prehospital electrocardiogram (ECG)’. Of these patient factors, three underlying categories were significantly associated with non-conveyance: ‘sudden cardiac death < 40 years of age in family history’ (OR 0.33, 95% CI 0.22–0.50; p < 0.001), ‘cardiovascular abnormalities, pulmonary embolism or pulmonary hypertension in the medical history’ (OR 0.62, 95% CI 0.43–0.91; p = 0.01), and ‘abnormal prehospital ECG’ (OR 0.54, 95% CI 0.41–0.72; p < 0.001).

**Conclusion:**

Sudden cardiac death < 40 years of age in family history, medical history, and abnormal ECG are significantly negatively associated with non-conveyance decision-making by EMS professionals in patients with a TLOC based on syncope. Low-risk factors do not play a significant role in conveyance decision-making.

**Supplementary Information:**

The online version contains supplementary material available at 10.1186/s12873-023-00890-y.

## Background

Emergency medical services (EMS) have reported a higher use of services over the past decades, increasing up to 39.8% over ten years [1–3]. With the general growth in EMS use, there is also an increase in the number of patients who receive ambulance care without conveyance. In the context of non-conveyance, the patient receives examination and treatment on scene, is discharged on scene, or may be referred to other healthcare facilities [4, 5]. Non-conveyance rates have been reported up to 30% [4].

Within the literature, patient safety in non-conveyance runs is reported with a diversity in outcomes. A systematic review showed that a significant amount of non-conveyed patients re-enter the emergency care chain [4]. After non-conveyance, 6.1% of the patients re-entered the EMS system within 24 h, and 19.0% visited an emergency department (ED) within 48 h. However, the data remained unclear whether the repeated EMS calls and ED visits were based on (new) medical necessity or if the complaints were similar to the initial EMS contact [4]. A Finnish cohort study showed that most non-conveyance patients had no adverse event [6]. If there was a re-contact with the emergency care chain, the complaint was often another one than the initial non-conveyance complaint [6].

The increase in ambulance usage and non-conveyance rates puts pressure on EMS professionals as they must make an independent clinical decision on whether or not to convey the patient to the ED. EMS professionals perceive the non-conveyance decision as complex and difficult [7]. It requires experience, knowledge, and dedication of the EMS professional [8]. In addition, conveyance decision-making can be influenced by factors of the system, such as the increasing demand for ambulance care, access to appropriate care options, disproportionate risk aversion, competencies, confidence and clinical training of the EMS professional, and preferences of the patient or his family [4, 9]. With regard to the competencies of EMS professionals, a meta-analysis showed insufficient evidence to support the determination of the medical necessity for ambulance transport by EMS professionals [10].

Within the non-conveyance population, a significant part of the patients have an initial emergency medical dispatch complaint or on-scene diagnosis that can be categorized as a transient loss of consciousness (TLOC) [11, 12]. TLOC is defined as a state of real or apparent loss of consciousness of short duration and is classified into four groups: syncope, epileptic seizure, psychogenic TLOC, and rare causes [13], of which syncope is the most common cause [13, 14]. Syncope is defined as a TLOC due to cerebral hypoperfusion. It is characterized by a rapid onset, short duration, and spontaneous complete recovery, and there are three underlying aetiology: reflex syncope, syncope due to orthostatic hypotension, and cardiac syncope [13]. The underlying aetiology of syncope determines the risk for short-term adverse events rather than the syncope itself [13, 15].

The European Society of Cardiology (ESC) provides a guideline with several patient factors to consider when estimating the underlying aetiology of a syncope [13]. These patient factors are categorised as determinants of the syncopal event, past medical history, physical examination, and electrocardiogram (ECG) and are determined through systematic patient assessment [13, 16]. Based on these patient factors, the EMS professional estimates the risk for short-term adverse events [16]. Conveyance decision-making in patients with syncope is perceived as extra complex due to the multiplicity of patient factors that can simultaneously be present in the patient and the limited information and available resources concerning decision-making [13, 14, 17, 18]. To advance risk stratification and decision-making, insight into patient factors that contribute to conveyance decision-making by EMS professionals is needed, as improved decision-making could lead to increased patient safety and more effective care at the right moment in the right place. The aim of this study was to explore patient factors significantly associated with conveyance decision-making by EMS professionals in patients with a TLOC based on syncope.

## Methods

### Study design

A cross-sectional factorial survey design using vignettes was conducted [19]. A vignette is a brief, written case history of a fictitious patient based on a realistic clinical situation [20, 21]. This method is suitable for uncovering respondents’ judgments of a situation and exploring factors and underlying categories in professionals’ clinical decision process [22]. This study was reported conform the STROBE statement [23].

### Setting and population

In the Netherlands, 25 EMS organisations provide prehospital emergency care, with 2483 clinically active EMS professionals [24]. Regular ambulances are staffed with a specially trained driver and an EMS professional. The EMS professional is trained in either the nursing or the medical domain. In the nursing domain, there is a specialised ambulance nurse (bachelor’s degree – NLQF 6) or a nurse practitioner (master’s degree - NLQF 7). Before completing the specialised degree in ambulance care, often, these bachelor nurses followed a specialisation in critical care, coronary care, hospital-based emergency care, or anaesthesiology [25]. Additionally, an 8-month specialised training with a final assessment is required to practice as an EMS professional [25]. In the medical domain, there is a professional with a Bachelor of Health (NLQF 6) or a physician assistant (master’s degree - NLQF 7). After the Bachelor of Health, the professional follows a nine to 12-month EMS traineeship, including assessment to become a registered EMS professional [26]. The route to both the nurse practitioner and the physician assistant is a master’s degree, following the Bachelor of Nursing or Bachelor of Health. In addition to working on regular ambulances, EMS professionals can act as rapid responders. The rapid responder works as a solo unit and can be dispatched when the medical dispatch centre, based on triage, expects the patient not to be conveyed [24].

Dutch EMS professionals work autonomously according to the ‘National Protocol Ambulance Care (LPA)’ and are supervised by an EMS physician responsible for medical care. The EMS supervisor is not present on scene but is permanently available for telephone consultation [27]. The Dutch National EMS umbrella organisation developed the LPA with medical experts to support clinical practice and decision-making. The protocols are updated regularly and consist of symptom-oriented flowcharts covering all aspects of prehospital emergency care [28]. These protocols are based on a mixture of evidence, best practice, and expert opinion [28]. Based on the LPA, the EMS professional decides on treatment and conveyance.

### Participants and recruitment

EMS professionals of all EMS organisations in the Netherlands were eligible to participate if they were.

- Registered as an EMS professional according to Dutch law;
- Clinically active within a Dutch EMS organisation;
- Involved in clinical decision-making in patients with TLOC.

Multiple methods were used to recruit participants. First, the researchers approached the professional organisation Dutch Ambulance Care Society (V&VN Ambulancezorg) and all the EMS organisations in the Netherlands (n = 25) via email. The organisations were asked to promote, distribute and invite respondents with a link and QR code to the questionnaire. Secondly, key figures with research affinity within EMS organisations were personally approached to recruit five or more EMS professionals. The questionnaire was available from October 19, 2020, to November 24, 2020.

### Modelling of the decision-making process

In order to provide insight into the conveyance decision-making process, we constructed vignettes with relevant patient factors for a TLOC based on syncope. The questionnaire and vignettes were developed for this study [see Additional file 1]. Patient factors related to syncope were derived from the protocol TLOC (syncope) of the LPA [16] [see Additional file 2] and were selected based on the European Society of Cardiology (ESC) guidelines for diagnosis and management of syncope [13]. Five patient factors were selected and incorporated in each vignette, presenting high or low-risk patient factors for adverse events (Table 1). Initially, the patient factors from the protocol TLOC (syncope) of the LPA consisted of two up to five underlying categories. We modified the underlying categories for two patient factors. For the patient factor ‘triggering factors’, the categories ‘fear’ and ‘emotion’ were merged into one category ‘emotion’. Fear was deemed a type of emotion, so it was not considered necessary to add it separately as a category. The categories of the patient factor ‘prehospital electrocardiogram (ECG)’ consisted of; ’normal ECG’, ‘rhythm and conduction disorder’, and ‘ischemia’. The categories ‘rhythm and conduction disorder‘ and ‘ischemia’ were merged into one category ‘abnormal ECG’, as the outcome of the conveyance decision-making process of both categories is the same. To determine patient factors to incorporate in the vignettes and modify the underlying categories, several experts were consulted. The consulted experts consisted of two nurse practitioners and two physician assistants specialised in ambulance care, two emergency physicians who were also EMS supervisors and two ambulance care professionals with a master’s degree in research. The research group made a first draft, which was tested with the experts through email or face-to-face consultation.

**Table 1:** Patient factors and categories in vignettes

Patient factor	Category (level)	Modified categories
Origin of event	a. During rest b. During exercise*	a. During rest b. During exercise*
Triggering factors	a. No triggering factors* b. Pain c. Fear d. Emotion(al) e. Prolonged standing	a. No triggering factors* b. Pain c. Emotion(al) d. Prolonged standing
Prodromal symptoms	a. No prodromal symptoms* b. Light headedness/dizziness c. Nausea, paleness, sweating d. Visual disturbances	a. No prodromal symptoms* b. Light headedness/dizziness c. Nausea, paleness, sweating d. Visual disturbances
Red flags	a. No red flags b. Sudden cardiac death < 40 years of age, in family history* c. Cardiovascular abnormalities, pulmonary embolism or pulmonary hypertension in medical history* d. First syncopal episode > 35 years of age*	a. No red flags b. Sudden cardiac death < 40 years of age, in family history* c. Cardiovascular abnormalities, pulmonary embolism or pulmonary hypertension in medical history* d. First syncopal episode > 35 years of age*
Prehospital ECG	a. Normal ECG b. Rhythm or conduction disorder on ECG* c. Ischemia on ECG*	a. Normal ECG b. Abnormal ECG*

* high-risk patient factor - abbreviations: ECG – electrocardiogram,

### Creation of vignettes

All possible combinations of patient factors and underlying categories combined, resulted in 256 unique vignettes (2*4*4*4*2 = 256). A standard vignette template was designed to prevent bias caused by different descriptions of the vignette [see Additional file 1: part two: vignettes] [20–22]. The template was a six-sentence-long scenario of a patient with a TLOC who is completely recovered when the ambulance arrives. The only varying component of the vignettes were the underlying categories of the patient factors. All 256 unique combinations of patient factors and underlying categories were inserted this way.

### Sample size calculation

The sample size calculation is based on the rule of 10 events per variable in the model [29]. An event was defined as the non-conveyance decision of the EMS professional. Our model included 11 degrees of freedom, and we estimated that 30% of the patients would not be conveyed based on international literature and national figures [4, 24, 27]. Therefore, 367 independent observations were needed. The sample size was corrected because of the hierarchical structure of our study (vignettes nested within respondents). Therefore, we assumed an intracluster correlation coefficient of 0.2. Based on 367 independent observations, 15 vignettes per respondent, an ICC of 0.2, an alpha of 0.05, and a power of 0.8, it was calculated that 93 respondents were needed. A statistician was involved in the sample size calculation.

### Data collection

Data was collected through an online questionnaire created in LimeSurvey (version 5.2.4). The survey consisted of two components. The first component consisted of respondent characteristics (i.e., gender, age, professional background, years of experience as an EMS professional, and EMS organisation). The second component consisted of a random sample of 15 vignettes. The randomisation of the vignettes was ensured within LimeSurvey so there would be an equal distribution of the underlying categories of the patient factors. For each vignette, the respondent had to indicate whether he would convey the patient to the hospital or decide to non-conveyance [see Additional file 1: part two: vignettes].

### Data analysis

The primary outcome measure was the percentage of vignettes with a non-conveyance decision of EMS professionals. First, data from LimeSurvey was prepared for data analysis. Respondents were excluded when they did not give consent or reported less than 14 vignettes. Descriptive statistics were performed to describe the study sample, i.e., number and percentages for categorical characteristics and mean and standard deviation for continuous characteristics. Because of the study’s data clustering, a multilevel logistic analysis was performed to model the probability of non-conveyance. Clustering appears for the vignettes and respondents; therefore, a model with a random intercept effect for vignettes and respondents and all other variables fixed was chosen. Univariate and multivariate multilevel logistic regression analysis was performed to identify associations between the factors and non-conveyance. Factors with a significance of p ≤ 0.20 in the univariate regression analysis were entered into a multilevel multivariate analysis. One at the time, non-significant variables were removed from the multivariate model until all variables were statistically significant. A p-value < 0.05 was considered statistically significant based on two-sided tests. Statistical analysis was developed and performed by a statistician using SPSS version 26.0 (IBM, in., Chicago, Il) and SAS/STAT software version 9.4.

### Ethics

This study was approved by the Research Ethics Committee of the HAN_ University of Applied Sciences, ECO 440.03/23. EMS professionals voluntarily participated in the study. Potential respondents received written information on the purpose of the study, data management, privacy aspects, and the required time investment. The questionnaire was not burdensome for respondents as the vignettes represented the daily work of an EMS professional and did not contain any questions that could cause potential psychological or emotional distress and were theoretical cases. After the provision of the study details, all EMS professionals provided informed consent.

## Results

### Study sample

In total, 178 EMS professionals responded to the survey. A total of 110 respondents were included. Sixty-eight respondents were excluded for not providing informed consent (n = 2), returning a blank survey (n = 16), and less than 14 completed vignettes (n = 50). A total of 1646 vignettes were assessed by respondents (106 respondents completed 15 vignettes, and four respondents completed 14 vignettes) and were taken in further analysis.

### EMS professional characteristics

Respondents from 14 out of the 25 Dutch EMS organisations were represented in the sample. The majority of the respondents were male (63.6%) with a mean age of 45.5 (± 9.3) years, see Table 2. The respondents’ most common EMS professional background was nursing (80%).

**Table 2:** Baseline characteristics of the EMS professionals

	EMS professional (n = 110)
Age in years, mean ± SD	45.5 ± 9.3
Male Gender, n (%)	70 (63.6%)
EMS Professional background, n (%) - Nursing - Bachelor of Health - Nurse Practitioner - Physician Assistant	88 (80.0%) 4 (3.6%) 11 (10.0%) 7 (6.4%)
Additional rapid responder training, n (%)	29 (26.4%)
Years of experience as EMS professional, mean ± SD	13.2 ± 8.9

### Vignettes

An equal distribution of the underlying categories of patient factors based on the number of categories per patient factor was seen after analysing all assessed vignettes (see Table 3). The patient factors with two underlying categories were distributed in approximately 50% of the vignettes, and the patient factors with four underlying categories appeared in approximately 25% of the vignettes. The EMS professionals decided to non-conveyance in 368 (22.4%) vignettes.

**Table 3:** Baseline characteristics of vignettes

Patient factor	Category (level)	Frequency
Total of vignettes, n		1646
Origin of event, n (%)	a. During rest b. During exercise	829 (50.4%) 817 (49.6%)
Triggering factors, n (%)	a. No triggering factors b. Pain c. Emotion(al) d. Prolonged standing	388 (23.6%) 377 (22.9%) 449 (27.3%) 432 (26.2%)
Prodromal symptoms, n (%)	a. No prodromal symptoms b. Light headedness/dizziness c. Nausea, paleness, sweating d. Visual disturbances	416 (25.3%) 405 (24.6%) 429 (26.1%) 396 (24.1%)
Red flags, n (%)	a. No red flags b. Sudden cardiac death < 40 years of age, in family history c. Cardiovascular abnormalities, pulmonary embolism or pulmonary hypertension in medical history d. First syncopal episode > 35 years of age	438 (26.6%) 421 (25.6%) 402 (24.4%) 385 (23.4%)
Prehospital ECG, n (%)	a. Normal ECG b. Abnormal ECG	840 (51.0%) 806 (49.0%)

Abbreviations: ECG – electrocardiogram

### Patient factors associated with conveyance decision-making

Univariate regression analysis showed that the patient factors ‘red flags’ and ‘prehospital ECG’ were significantly associated with conveyance decision-making. Of the patient factor ‘red flags’, particularly the underlying categories ‘sudden cardiac death < 40 years of age in family history’ (OR 0.32, 95%CI 0.21–0.50; p < 0.001) and ‘cardiovascular abnormalities, pulmonary embolism or pulmonary hypertension in medical history’ (OR 0.61, 95% CI 0.41–0.90; p = 0.01) were associated with a decreased likelihood of non-conveyance. This association was also present with the patient factor ‘prehospital ECG’, where the underlying category ‘abnormal prehospital ECG’ showed an OR 0.55 (95% CI 0.41–0.74; p < 0.001) for non-conveyance, see Table 4.

**Table 4:** Univariate and multivariate regression analysis of variables associated with non-conveyance

			Univariate analysis		Multivariate analysis	
Independent variable	**Treated on scene**	**Transported to ED**	**OR (95% CI)**	**P value**	**OR (95% CI)**	**P value**
*Origin of event, n (%)*						
During rest (ref) During exercise	200 (24%) 168 (21%)	629 (76%) 649 (79%)	0.82 (0.61–1.11)	0.20		
*Triggering factors, n (%)*				0.24		
No trigging factors (ref) Pain Emotion(al) Prolonged standing	70 (18%) 81 (21%) 109 (24%) 108 (25%)	318 (82%) 296 (79%) 340 (76%) 324 (75%)	1.28 (0.82–2.01) 1.46 (0.95–2.25) 1.50 (0.98–2.32)	0.27 0.08 0.06		
*Prodromal symptoms, n (%)*				0.60		
No prodromal symptoms (ref) Light-headedness/dizziness Nausea, paleness, sweating Visual disturbances	93 (22%) 100 (25%) 97 (23%) 78 (20%)	323 (78%) 305 (75%) 332 (77%) 318 (80%)	1.11 (0.73–1.69) 1.04 (0.68–1.58) 0.83 (0.54–1.28)	0.63 0.87 0.40		
*Red flags, n (%)*				< 0.001		< 0.001
No red flags (ref) Sudden cardiac death < 40 years of age, in family history Medical history of cardiovascular abnormalities, pulmonary embolism or pulmonary hypertension First syncopal episode > 35 years of age	129 (29%) 49 (12%) 79 (20%) 111 (29%)	309 (71%) 372 (88%) 323 (80%) 271 (71%)	0.32 (0.21–0.50) 0.61 (0.41–0.90) 1.10 (0.76–1.62)	< 0.001 0.01 0.61	0.33 (0.22–0.50) 0.62 (0.43–0.91) 1.14 (0.79–1.63)	< 0.001 0.01 0.49
*Prehospital ECG, n (%)*						
Normal ECG (ref) Abnormal ECG	230 (27%) 138 (17%)	610 (73%) 668 (83%)	0.55 (0.41–0.74)	< 0.001	0.54 (0.41–0.72)	< 0.001

Abbreviations: ECG – electrocardiogram

In the multivariate regression analysis, the patient factors ‘red flags’ and ‘prehospital ECG’ remained significantly associated with conveyance decision-making. Of these patient factors, the three underlying categories ‘sudden cardiac death < 40 years of age in family history’ (OR 0.33, 95%CI 0.22–0.50; p < 0.001), ‘cardiovascular abnormalities, pulmonary embolism or pulmonary hypertension in the medical history’ (OR 0.62, 95% CI 0.43–0.91; p = 0.01), and ‘abnormal prehospital ECG’ (OR 0.54, 95% CI (0.41–0.72; p < 0.001) remained associated with a decreased likelihood of non-conveyance.

## Discussion

In a modelled study setting, this study aimed to identify patient factors significantly associated with conveyance decision-making in patients with a TLOC based on syncope by EMS professionals. Multivariate regression analysis showed that the patient factors ‘red flags’ and ‘prehospital ECG’ contributed significantly to conveyance decision-making. The underlying categories of ‘red flags’ (‘sudden cardiac death < 40 years of age in family history’ and ‘cardiovascular abnormalities, pulmonary embolism or pulmonary hypertension in the medical history’) and ‘prehospital ECG’ showed an odds ratio < 1, meaning it is more likely that the patient will be conveyed to the hospital if one of these categories is present. These results show that cardiac risk factors appear to play a significant role in conveyance decision-making.

This study demonstrates that conveyance decision-making is mainly based on high-risk patient factors. The presence of a high-risk patient factor significantly reduces the likelihood of a non-conveyance decision. Our data did not reveal that low-risk patient factors significantly influence conveyance decision-making, although low-risk patient factors are associated with a lower risk of short-term serious adverse events [13, 17]. It is possible that high-risk patient factors might have such an influence on the risk stratification of EMS professionals that any effects of low-risk patient factors are canceled out. EMS professionals’ emphasis on high-risk patient factors might reflect their commitment to ensuring patient safety and, thereby, possibly a predisposition to convey patients to the ED to avoid any risk. The EMS professional’s current professional structure also focuses on this because the national guideline mainly focuses on high-risk patient factors [16]. However, the current ESC guidelines also describe a number of low-risk patient factors that are also important in risk stratification [13]. Potentially, a risk stratification tool for patients with a TLOC based on syncope in EMS, including low-risk factors, could aid the EMS professional in their conveyance decision-making process. This could lead to a safe and enhanced prehospital conveyance decision and decreased ED presentations of patients with a TLOC based on syncope with low-risk patient factors. This seems relevant since a Canadian cohort study revealed that 76.3% of all syncope patients conveyed to the ED were discharged immediately [30]. These discharged patients were considered low-risk and rarely showed short-term serious adverse events [30]. However, the current literature does not describe such an instrument.

Furthermore, our results showed that the prehospital ECG contributed significantly to the conveyance decision, e.g. the referral of the patient to the ED. In our study, the interpretation of the prehospital ECG was already provided in the vignettes. Therefore, participants did not have to assess the ECG abnormalities by themselves. It has been reported that (EMS) nurses have deficiencies in ECG interpretation skills [31, 32]. This raises the question of whether EMS professionals working in prehospital emergency care are able to interpret and recognise prehospital ECG abnormalities in real life (next to the patient) and, furthermore, make an appropriate conveyance decision. In addition, in syncope patients, ECG interpretation is extra complicated, as syncope patients could have rare but specific and difficult-to-assess ECG abnormalities such as ventricular hypertrophy, cardiomyopathy, long QT-, Brugada-, or Wolff-Parkinson-White syndrome [13, 17]. Because these abnormalities are difficult to detect and interpret, it makes the decision-making process even more difficult. Additionally, as EMS professionals are insufficiently exposed to these specific ECG abnormalities in practice, it is possible that EMS professionals do not adequately recognise them [31]. Another difficulty is that these ECG abnormalities already have normalised when the EMS professional makes the prehospital ECG. Nevertheless, these abnormalities are all associated with a high risk of short-term serious adverse events [13, 17]. Therefore, continuous education of EMS professionals on case presentations and assessment of ECG abnormalities in prehospital emergency care remains necessary.

Our vignettes were based on a national guideline [16], and the results show that EMS professionals are familiar with this guideline as conveyance decision-making was mainly based on high-risk patient factors, similar to the focus of the national guideline. However, the vignettes did not fully represent the complex real work environment of EMS professionals. For example, the vignettes contained mainly medical factors and did not include many contextual factors, although it has been reported earlier that contextual factors play a role in the decision-making process of the EMS professional [33]. The decision-making process is further influenced by factors such as personal identification. This means that an EMS professional identifies him- or herself with (the situation of) a patient [26]. Furthermore, the social context of the patient can influence the decision-making process of EMS professionals. When social support of the patient or a district nurse is present on scene, EMS professionals are more likely to decide to non-conveyance of the patient [34]. Also, personal attitudes towards non-conveyance of the patient and their relatives can influence the decision-making process [35, 36]. Non-medical factors, such as contextual factors, personal identification, and the patient’s social situation, were not simulated due to methodological reasons. When non-medical factors were also included in this study, the number of factors and, likewise, the number of respondents increased so much that effective recruitment was impossible.

A limitation of the vignette study is the theoretical nature of its design. Although the vignettes were based on international and national guidelines and checked by an expert panel, it is a simulation and simplification of the real world. Participants performed their decision-making process in front of an electronic device. However, all the created vignettes had a standardised design, which reduced bias. In total, n = 66 respondents completed less than 14 vignettes; this could be related to the fact that respondents filled in the questionnaire during their shift and had to quit when they were called for an emergency ride.

It might be possible that the convenience sampling method introduced selection bias, mainly for master-educated EMS professionals. In the Netherlands, 80 nurse practitioners and physician assistants are employed in EMS care [37]. In our study, n = 18 (22.5%) master-educated EMS professionals in the Netherlands responded, which seems to be an overrepresentation of this group in our study. Furthermore, it is possible that EMS professionals who participated in this study were enthusiastic about TLOC and syncope and interested in this topic. Therefore, they could have had more knowledge of TLOC and syncope compared to other EMS professionals who did not participate in the study. Nevertheless, the baseline characteristics of the participating bachelor EMS professionals (e.g. distribution of participating EMS organisations, age, and years of experience as EMS professional) seem to adequately represent the EMS population in the Netherlands [24]. Finally, this study was conducted in the Netherlands, where the EMS system and the educational level of EMS professionals differ from other countries. This might limit the generalisability of our results to other countries with different healthcare or educational systems.

Despite these limitations, we feel that this study provided a relevant and interesting insight into the decision-making processes in TLOC patients based on syncope of EMS professionals. Patient factors that are associated with conveyance decision-making in TLOC patients were identified. Future research should further explore and focus on identifying non-medical factors and how these factors influence conveyance decision-making of EMS professionals in TLOC patients based on syncope. Non-medical factors could affect the identified patient factors and, therefore, could be potential confounders. In this line of reasoning, the results of this study could be overestimated. Additionally, evidence-based tools should be developed to aid the EMS professional in the risk assessment of patients with a TLOC based on syncope and to guide the EMS professional in the conveyance decision-making process.

## Conclusions

This study provided insights into the complex conveyance decision-making of EMS professionals in patients with a TLOC based on syncope. Our study indicated that cardiac high-risk patient factors were significantly associated with conveyance decision-making. The underlying categories ‘sudden cardiac death < 40 years of age in the family history’, ‘medical history’, and ‘abnormal prehospital ECG’ were associated with a decreased likelihood of non-conveyance by EMS professionals in patients with a TLOC based on syncope. Low-risk patient factors do not play a significant role in conveyance decision-making. Future studies should also include non-medical factors in risk stratification and decision-making in patients with a TLOC based on syncope, as these could have a relevant (confounding) effect.

### Electronic supplementary material

Below is the link to the electronic supplementary material.

Supplementary Material 1

Supplementary Material 2

## Data Availability

The dataset generated and analysed during the current study is not publicly available due to the informed consent procedure which did not explicitly include open access data sharing. The data will be made available from the corresponding author for the editor/reviewers upon request.

## Abbreviations

ED: Emergency Department
EMS: Emergency Medical Services
ECG: Electrocardiogram
TLOC: Transient Loss of Consciousness
LPA: ‘National Protocol Ambulance Care’ (in Dutch: Landelijk Protocol Ambulancezorg)
