# Supplementary material for: Patient factors associated with conveyance decision-making by Emergency Medical Services professionals in patients with a syncope: a cross-sectional factorial survey design
Source: BMC Emerg Med. 2023 Oct 5;23:118. doi: 10.1186/s12873-023-00890-y (PMC10557231; doi:10.1186/s12873-023-00890-y)
Supplement: Supplementary file 1 — Supplementary Material 1 [file 12873_2023_890_MOESM1_ESM.docx]

**Additional file 1**

Questionnaire and vignettes developed for this study translated in English. **Part one: general demographic data** consist of the baseline data. **Part two: Vignette study** consists of five examples of the presented vignettes, the framework for the vignettes, and the variables used.

**Questionnaire**

Dear EMS professional,

Thank you for taking the time to complete this survey. This survey is part of the SAFE END project. The SAFE END project is based on previous research that shows that risk assessment and consideration of choices regarding non-conveyance is a challenge for EMS professionals. Furthermore, the theme of non-conveyance is ranked at number one in the Dutch National EMS research agenda. The most common initial complaint in non-conveyance is a transient loss of consciousness (syncope). Transient loss of consciousness, as you know, is a complex complaint due to its various etiology/causes. The SAFE END project develops tools to support risk assessment and decision-making to treat on-site or to transport the patient with a transient loss of conscious to the emergency department. With these tools, we aim to increase patient safety by preventing unnecessary referrals or a delay in necessary referrals to the emergency department.

To ensure that these tools meet the needs of EMS professionals, we are curious about your experiences and competencies regarding patients with a transient loss of consciousness (syncope). This survey is entirely anonymous and will be used by the researchers to identify areas for improvement to guide decision-making. The results of this survey are not personally identifiable and will not be shared with your employer or others. The data is stored anonymously on a secure data disk at the HAN University of Applied Sciences. Only the researchers have access to this data. If you wish, we will send you a report of the results. You can enter your e-mail address for this purpose at the end of the questionnaire. The survey will take 20-30 minutes of your time.

If you have any questions, please feel free to contact Bastiaan Ort via [bastiaan.ort@han.nl](mailto:bastiaan.ort@han.nl)

Kind regards,

Sivera Berben, project manager SAFE END study & associate professor, HAN University of Applied Sciences

Bastiaan Ort, junior researcher SAFE END study, HAN University of Applied Sciences

**I agree to participate in this research and have been sufficiently informed.**

*Information entered in this questionnaire will remain anonymous. There is no judgment on the data you enter as an ambulance care professional. This data is used for research purposes only.*

- Yes
- No

**Part one: general demographic data**

**1. What is your age?**

________________

**2. What is your gender?**

- Male
- Female

**3. What is your professional background?**

- Nursing
- Bachelor of Health
- Nurse Practitioner
- Physician Assistant

**4. I also work as a soloist/rapid responder:**

- Yes
- No

**5. Which EMS organisation do you work for?**

- Ambulancezorg Groningen
- UMC Ambulancezorg
- RAV Fryslân
- RAV IJsselland/Ambulance Oost
- Witte Kruis
- Veiligheids- en Gezondheidsregio Gelderland-Midden
- RAV Gelderland Zuid
- RAVU
- RAV Noord-Holland Noord
- RAV Kennemerland
- Ambulance Amsterdam
- RAV Flevoland/Gooi en Vechtstreek
- RAV Haaglanden
- RAV Hollands Midden
- AmbulanceZorg Rotterdam-Rijnmond
- Veiligheidsregio Zuid-Holland Zuid
- RAV Brabant Midden-West-Noord
- GGD Brabant-Zuidoost
- AmbulanceZorg Limburg-Noord
- GGD Zuid Limburg

**6. How many years of work experience do you have within the EMS?**

_____________

**Part Two-: Vignette study**

Ten different case studies will be presented. Each case study consists of a patient description There are two decision options at the end of each case study. Would you like to transport this patient to the hospital for further examination and treatment of the syncope: then choose “transport to hospital”. If you think further examination into the cause of the syncope is not necessary and on-site treatment will be sufficient and adequate, than you can choose “non-conveyance”. You may wish to have more information before making a decision, however we do not provide additional information. In this cases gender is not considered to be relevant for the decision-making. We advise you to make brief clinical reasoning assessment based on the available information and to make a decision that seems appropriate for the presented patient at this time.

*Examples of case studies presented:*

**Case study**: Patient X. had a transient loss of consciousness (TLOC) during excersise. He was in pain at the time of TLOC and regained full consciousness within 15 minutes. He felt completely normal upon arrival of the ambulance. An anamnesis is taken by the EMS professional, and the following findings are discovered: before he became unconscious, he briefly had a feeling of light-headedness. His family history shows occurrence of sudden cardiac death < 40 years of age. Upon the ECG, the following 12 leads ECG is seen: a sinus rhythm.

**Case study**: Patient X. had a transient loss of consciousness (TLOC) during excersise. He had no symptoms at the time of TLOC and regained full consciousness within 15 minutes. He felt completely normal upon arrival of the ambulance. An anamnesis is taken by the EMS professional, and the following findings are discovered: before he became unconscious, he had no symptoms. His history shows cardiovascular abnormalities. Upon the ECG, the following 12 leads ECG is seen: ischemia.

**Case Study**: Patient X. had a transient loss of unconsciousness (TLOC) during rest. He had been standing for a long time at that time of TLOC and regained full consciousness within 15 minutes. He felt completely normal upon arrival of the ambulance. An anamnesis is taken by the EMS professional, and the following findings are discovered: before he became unconscious, he briefly had feelings of nausea, paleness, and sweating. His history shows this is his first syncopal episode <35 years. Upon the ECG, the following 12 leads ECG is seen: a sinus rhythm.

**Case Study**: Patient X. had a transient loss of consciousness (TLOC) during rest. He was experiencing emotion at the time of TLOC and regained full consciousness within 15 minutes. He felt completely normal upon arrival of the ambulance. An anamnesis is taken by the EMS professional, and the following findings are discovered: before he became unconscious, he briefly had a visual disturbance. His history shows cardiovascular abnormalities. Upon the ECG, the following 12 leads ECG is seen: ventricular tachycardia.

**Case Study**: Patient X. had a transient loss of consciousness (TLOC) during exercise. He had been standing for a long time at that time of TLOC and regained full consciousness within 15 minutes. He felt completely normal again upon arrival of the ambulance. An anamnesis is taken by the EMS professional, and the following findings are discovered: before he became unconscious, he had brief feelings of nausea, paleness, and sweating. Both his history and that of his family are blank. Upon the ECG, the following 12 leads ECG is seen: a sinus rhythm.

*Framework case studies*

**Case Study**: Patient X. had a transient loss of consciousness (TLOC) during [**insert variable A**]. He had [**insert variable B**] at that time of TLOC and regained full consciousness within 15 minutes. He felt completely normal upon arrival of the ambulance. The medical history is taken by the EMS professional, and the following findings are discovered: before he became unconscious, he had [**insert variable C**]. His history shows [**insert variable D**]. Upon the ECG, the following 12 leads ECG is seen: [**insert variable E**].

| **Variables** | **Category** |
| --- | --- |
| Variable A – origin of event | - During rest - During exercise |
| Variable B – triggering factors | - Pain - Emotion - Prolonged standing - No triggering factors |
| Variable C – prodromal symptoms | - No prodromal symptoms - Light headedness/dizziness - Nausea, paleness, sweating - Visual disturbances |
| Variable D – red flags | - No red flags - Sudden cardiac death <40 years of age, in family history - Cardiovascular abnormalities, pulmonary embolism or pulmonary hypertension in medical history - First syncopal episode < 35 years of age |
| Variable E – prehospital ECG | - Normal ECG: sinus rhythm - Abnormal ECG* |

*To make the vignettes correspond to real-life situations, abnormal ECG was translated into the vignettes as the following ECG abnormalities, based on the ESC-guidelines: ischemia, 3^e^ degree AV block, atrial fibrillation (<40 b.p.m.) of ventricular tachycardia
