# Supplementary material for: Patient factors associated with conveyance decision-making by Emergency Medical Services professionals in patients with a syncope: a cross-sectional factorial survey design
Source: BMC Emerg Med. 2023 Oct 5;23:118. doi: 10.1186/s12873-023-00890-y (PMC10557231; doi:10.1186/s12873-023-00890-y)
Supplement: Supplementary file 2 — Supplementary Material 2 [file 12873_2023_890_MOESM2_ESM.pdf]

Additional file 2: Translation of the National Protocol Ambulance Care (LPA) 8.1

Protocol 4.5 – Transient loss of consciousness (syncope)

4.5 Transient loss of consciousness (syncope)

Sudden transient loss of consciousness
with rapid spontaneous full recovery

History of the event

- Triggering factor(s)*
- Prodromal symptoms **
- Briefly unconscious
- Clear consciousness immediately after transient loss of consciousness
- No complaints within 15 minutes

Rule-out

- ECG: ischemia, rhythm/conduction disorders
- Syncope during exercise
- Neurological symptoms/abnormalities
- Blood glucose level < 3,5 mmol/l
- Red flags ***

** Triggering factors*

- *Emotional stimulus, fear, pain*
- *Prolonged standing in crowded, hot environment*

*** Prodromal symptoms*

- *Light headedness/dizziness*
- *Nausea, paleness, sweating*
- *Visual disturbances*

**** Red flags*

- *Sudden cardiac death < 40 years of age, in family history*
- *1st episode > 35 years of age*
- *Medical history: cardiovascular abnormalities, pulmonary embolism, pulmonary hypertension*

in ’t Veld C, van Exter P, Rombouts M, de Visser M, de Vos R, Lelieveld K, et al. Landelijk Protocol Ambulancezorg - Versie 8.1 [Internet]. Zwolle: Ambulancezorg Nederland; 2016. 1–288 p. Available from: https://www.ambulancezorg.nl/static/upload/raw/d24e66de-8f05-47a1-92cd-3ec0278c2287/Ambulancezorg_Nederland_LPA8.1_herdruk_2019.oktober.wm.bv.pdf
